# A Thermodynamic Model for Interpreting Tryptophan Excitation-Energy-Dependent Fluorescence Spectra Provides Insight Into Protein Conformational Sampling and Stability

**DOI:** 10.3389/fmolb.2021.778244

**Published:** 2021-12-03

**Authors:** A Kwok, IS Camacho, S Winter, M Knight, RM Meade, MW Van der Kamp, A Turner, J O’Hara, JM Mason, AR Jones, VL Arcus, CR Pudney

**Affiliations:** ^1^ Department of Biology and Biochemistry, University of Bath, Bath, United Kingdom; ^2^ Biometrology, Chemical and Biological Sciences Department, National Physical Laboratory, London, United Kingdom; ^3^ UCB, Slough, United Kingdom; ^4^ School of Biochemistry, University of Bristol, Bristol, United Kingdom; ^5^ School of Science, Faculty of Science and Engineering, University of Waikato, Hamilton, New Zealand; ^6^ BLOC Laboratories Limited, Bath, United Kingdom

**Keywords:** protein stability, red edge excitation shift, fluorescence, tryptophan, conformational sampling

## Abstract

It is now over 30 years since Demchenko and Ladokhin first posited the potential of the tryptophan red edge excitation shift (REES) effect to capture information on protein molecular dynamics. While there have been many key efforts in the intervening years, a biophysical thermodynamic model to quantify the relationship between the REES effect and protein flexibility has been lacking. Without such a model the full potential of the REES effect cannot be realized. Here, we present a thermodynamic model of the tryptophan REES effect that captures information on protein conformational flexibility, even with proteins containing multiple tryptophan residues. Our study incorporates exemplars at every scale, from tryptophan in solution, single tryptophan peptides, to multitryptophan proteins, with examples including a structurally disordered peptide, *de novo* designed enzyme, human regulatory protein, therapeutic monoclonal antibodies in active commercial development, and a mesophilic and hyperthermophilic enzyme. Combined, our model and data suggest a route forward for the experimental measurement of the protein REES effect and point to the potential for integrating biomolecular simulation with experimental data to yield novel insights.

## Introduction

Tracking protein conformational change and, even more subtly, changes in the equilibrium of available conformational states is central to molecular biosciences. Protein stability is intimately linked with the distribution of conformational states (Karshikoff et al., 2015) and, as a good generalization, increased stability tracks with a decrease in the distribution of conformational states (increasing rigidity, decreasing conformational entropy). (Vihinen, 1987). While engineering protein stability has advanced enormously, the tools to sensitively and quantitatively track these changes are lacking. There are a broad range of potential analytical tools, but only a few that can be applied routinely to the vast majority of proteins without unreasonable requirements regarding solvent, protein concentrations, and thermal stability, or without the requirement of surface attachment or labeling. (Magliery et al., 2011). Moreover, the vast majority of protein conformational changes are subtle, described as “breathing” motions, where most structural orders (primary to quaternary) of the protein are not altered, but it is the equilibrium of conformational states (protein flexibility) that changes (Kossiakoff, 1986).

**FIGURE 1:**
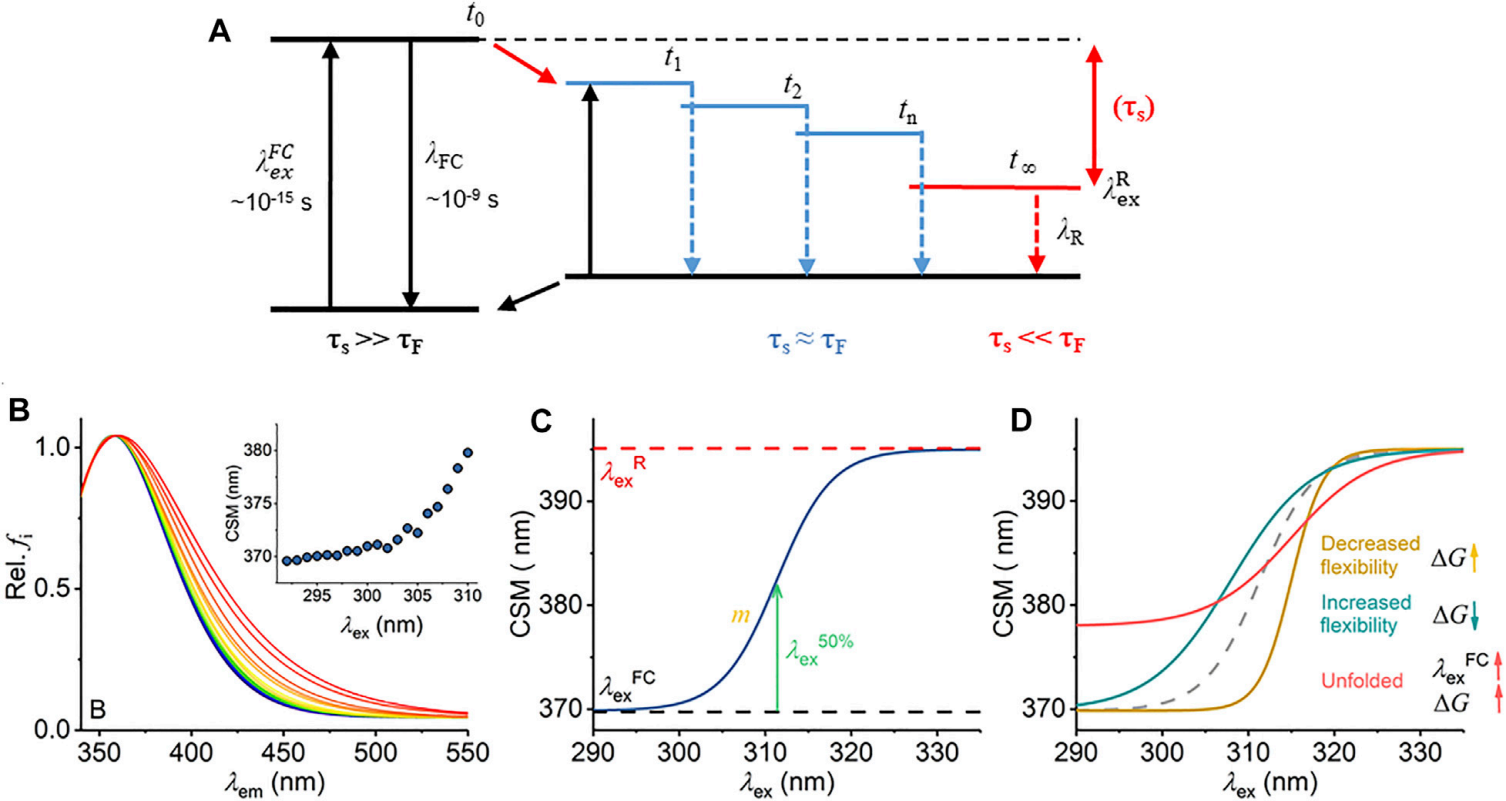
Mechanism of the REES effect, predicted experimental observables, and graphical description of Eq. (7). **(A)** Jablonski diagram illustrating the REES effect. **(B)** Example model Trp REES data showing the normalized emission spectrum with increasing excitation wavelength and inset as the change in CSM versus excitation wavelength. **(C)** Graphical depiction of Eq. (7). **(D)** Predicted spectral changes resulting from variations in Eq. (7) from shifts in protein flexibility and conformational state (folding).

The red edge excitation shift (REES) phenomenon is a sensitive reporter of a fluorophore’s environment, and the mechanism is shown in Figure 1A. (Azumi and Itoh, 1973; Itoh and Azumi, 1975; Azumi et al., 1976; Demchenko, 2002). Briefly, the REES effect is sensitive to shifts in the distribution of environments a fluorophore can sample. As this distribution of environments gets smaller, the REES effect becomes “smaller” (we discuss this in depth below) and vice versa. In proteins, such shifts in the distribution of states are how we conceptualize protein motions. Potentially then, the REES effect could be a powerful tool, both to study protein flexibility/motion but also (as above) stability.

Radiative fluorescence takes place after light absorption alongside non-radiative processes, which include vibrational relaxation and solvent relaxation (dipolar re-organization). Vibrational relaxation is typically fast (∼10^−12^ s) relative to the lifetime of fluorescence emission (*τ*_F_ ∼ 10^−10^–10^−9^ s) and so causes a complete relaxation of the system to its lowest energy level prior to emission. This gives rise to the familiar red shift of fluorescence emission compared to absorption (Stokes shift). The Lippert–Mataga equation [Eq. (1)] illustrates that the greater the polarity of solvent, the larger the anticipated Stokes shift (Mataga et al., 1956; Lippert Von, 1957).

$${\bar{v}}_{A}-{\bar{v}}_{E}=\frac{2}{hc}\left(\frac{\varepsilon -1}{2\varepsilon +1}-\frac{n^{2}-1}{2n^{2}+1}\right)\frac{{\left(\mu_{E}-\mu_{G}\right)}^{2}}{a^{3}}+L$$ (1)

where the Stokes shift (difference between wavenumber of absorption and emission), ${\bar{v}}_{A}-{\bar{v}}_{E},$ is governed by the dielectric constant of the solvent, *ε*, specifically the reorientation of solvent dipoles; the refractive index, *n*; the dipole moment of the ground and excited states, $\mu_{G}$ and $\mu_{E}$ , respectively; the radius of the fluorophore cavity, *a*; and a constant, *L*.

Equation (1) assumes that the solvent relaxation is complete prior to emission. However, solvent relaxation is not necessarily always fast relative to fluorescence emission and under a range of solvent or environmental conditions can approach *τ*_F_ [∼10^−10^ –10^−9^ s]. The longer solvent relaxation lifetime (*τ*_S_) can therefore affect the level from which emission occurs and so the emission wavelength, in which case it also contributes to the Stokes shift (Azumi and Itoh, 1973; Itoh and Azumi, 1975; Azumi et al., 1976; Demchenko, 2002). Specifically, one expects that an ensemble of energetic substates is formed related to the distribution of solvent relaxation lifetimes, i.e., the available distribution of solvent–fluorophore interaction energies. The additive contribution of these states to the steady-state emission spectrum gives rise to broad-band emission, which is observed as inhomogeneous broadening of the spectra. This broadening is then dependent on the excitation energy used, since as one decreases the excitation energy, there is an increasing photoselection of states (Figure 1A). Experimentally, one then observes a red shift in the emission spectra with respect to increasing excitation wavelength, i.e., decreasing excitation energy (Figure 1B). The inhomogeneous broadening will be dependent on a range of physical conditions that affect τ_S_, including temperature, viscosity, and solvent dipole moment (and therefore the solvent dielectric constant) (Azumi and Itoh, 1973; Itoh and Azumi, 1975; Azumi et al., 1976; Demchenko, 2002).

The sensitivity of the REES effect to changes in the equilibrium of solvent–fluorophore interaction energies suggests potential for using the approach to track changes in protein conformational state using the intrinsic fluorescence of the aromatic amino acids (Demchenko, 2002; Chattopadhyay and Haldar, 2014). Indeed, tryptophan (Trp) has been shown to give a large REES effect in numerous proteins, and we point to excellent reviews that illustrate key examples (Demchenko and Ladokhin, 1988; Raghuraman et al., 2005; Chattopadhyay and Haldar, 2014; Brahama and Raghuraman, 2021). Demchenko and Ladokhin (1988) suggest that the selection between ^1^L_a_ and ^1^L_b_ electronic excited states of Trp acts to increase the magnitude of the red edge excitation shift. Trp has the advantage that its emission can be separated from tyrosine (Tyr) and phenylalanine (Phe) by excitation at wavelengths >∼292 nm (Adman and Jensen, 1981). Trp REES is therefore a potentially excellent probe of protein conformational change, intrinsic disorder, and possibly even of changes in the equilibrium of conformational states.

We have previously applied and validated an empirical model for describing protein REES data as a function of the equilibrium of conformational states, which we call QUBES (quantitative understanding of biomolecular edge shift) (Catici et al., 2016; Jones et al., 2017; Knight et al., 2020). Herein, we refer to changes in the equilibrium of conformational states as changes in flexibility, with a more flexible protein having a broader equilibrium of conformational states. We track the changes in inhomogeneous broadening as the change in the center of spectral mass (CSM; Eq. (2)) of steady-state emission spectra (example shown in Figure 1B).

$$CSM=\;\frac{\sum \left(f_{i}\times \lambda_{Em}\right)}{\sum \left(f_{i}\right)}$$ (2)

where *f*_*i*_ is fluorescence intensity, and $\lambda_{Em\;}$ is the emission wavelength. The resulting data are then fit to the QUBES model [Eq. (3)].

$$CSM=CSM_{0}+Ae^{R\text{Δ}\lambda_{Ex}}$$ (3)

where $CSM_{0}$ is the CSM value independent of $\lambda_{Em\;}$ , and $\lambda_{Ex\;}$ is the excitation wavelength. The amplitude relative to $CSM_{0}$ and curvature of the exponential are described by *A* and *R*, respectively. We have previously found that the parameters from this empirical model could be used to track changes in protein stability (Jones et al., 2017; Knight et al., 2020; Hindson et al., 2021). That this simple model appears to provide useful insight suggests that it is approximating the protein REES effect to a level of accuracy.

While Eq. (3) performs well at tracking shifts in protein rigidity/flexibility (also for multi-Trp containing proteins) (Catici et al., 2016; Jones et al., 2017; Knight et al., 2020; Hindson et al., 2021), it does not relate to a specific thermodynamic parameter and neglects the fact that protein Trp emission will have a finite maximum observable spectral red shift at $\lambda_{ex}^{R}$ . Moreover, the data from our QUBES model cannot be cross-compared to proteins with different Trp content and location in structure. Developing our QUBES model towards an accurate *a priori* thermodynamic model would therefore enhance both the accuracy and utility of the approach for studying protein dynamics/stability.

Herein, we describe a thermodynamic model for interpreting protein REES data, which builds on our early work. Using a range of model systems from Trp/solvent studies, single Trp-containing proteins and multi-Trp proteins, we find that the new model accurately tracks with independent metrics of changes in the equilibrium of protein conformational states and more gross metrics of protein folding. Moreover, our model points to the need for new experimental approaches to monitor the protein REES effect.

## Results and Discussion

As described by Demchenko and Ladokhin (1988), we posit a two-state model and assume [FC]⇌[R] and $\tau_{F}\ll \tau_{S}$ ; then, the fractional concentration of *R* is given by:

$$\frac{\left[R\right]}{\left[FC\right]+\left[R\right]}=\frac{e^{\frac{-\Delta G}{RT}}}{1+e^{\frac{-\Delta G}{RT}}}$$ (4)

where ∆*G* is the difference in free energy between the [*FC*] (Frank–Condon) and [*R*] (relaxed) states, noting that the *RT* term is gas constant temperature. We then assume that ∆*G* will change linearly with excitation wavelength:

$$\Delta G=\Delta G_{\lambda_{ex}^{FC}}-m\Delta \lambda_{ex}$$ (5)

with a gradient, *m*. Thus, we anticipate a two-state transition between *FC* and *R* states due to photoselection by excitation wavelength with baselines $CSM\left(\lambda_{ex}^{FC}\right)$ and $CSM\left(\lambda_{ex}^{R}\right)$ , respectively. The gradient of the transition is given by |∆*G*| at any particular $\lambda_{Ex\;}$ .

$$CSM\left(\lambda_{ex}\right)=\frac{CSM\left(\lambda_{ex}^{FC}\right)+CSM\left(\lambda_{ex}^{R}\right)e^{\frac{-\Delta G}{RT}}}{1+e^{\frac{-\Delta G}{RT}}}$$ (6)

$$CSM\left(\lambda_{ex}\right)=\frac{CSM\left(\lambda_{ex}^{FC}\right)+CSM\left(\lambda_{ex}^{R}\right)e^{m\left(\lambda_{ex}-\lambda_{ex}^{50\text{\%}}\right)/RT}}{1+e^{m\left(\lambda_{ex}-\lambda_{ex}^{50\text{\%}}\right)/RT}}$$ (7)

Equations (6) and (7) establish three key parameters, $CSM\left(\lambda_{ex}^{FC}\right)$ , $CSM\left(\lambda_{ex}^{R}\right)$ , and Δ*G*, which we describe below. Figure 1C shows Eq. (7) plotted in a similar manner to the experimental data as in Figure 1B inset but now showing the full range of the function. Equation (7) is a more complete description of the REES effect [c.f. Eq. (3)] since it predicts a maximum magnitude of the CSM, corresponding to the fully relaxed state, $\lambda_{ex}^{R}$ (Figure 1C). Clearly, the emission spectra cannot become infinitely inhomogeneously broadened, and so the REES effect must saturate. Indeed, we and others have observed saturation of the REES effect with non-Trp fluorophores used as molecular probes (Gulácsy et al., 2019) or ligands (Kabir et al., 2021), and so Eq. (7) is logical for the REES effect in proteins. $CSM\left(\lambda_{ex}^{FC}\right)$ is the CSM corresponding to $\lambda_{ex}^{FC}$ shown in Figure 1A. We anticipate that $CSM\left(\lambda_{ex}^{FC}\right)$ will be responsive to changes in solvation environment in a similar way to the spectral shift of Trp on solvent/exposure/occlusion. That is, as the Burstein classification (Reshetnyak et al., 2001) and Eq. (1), increasing solvent exposure will cause $CSM\left(\lambda_{ex}^{FC}\right)$ to red shift and a decrease in solvent exposure will cause $CSM\left(\lambda_{ex}^{FC}\right)$ to blue shift (Reshetnyak et al., 2001).

$CSM\left(\lambda_{ex}^{R}\right)$ is the CSM corresponding to $\lambda_{ex}^{R}$ in Figure 1A, i.e., the completely relaxed state of the solvent. Note that this value should be fixed for a given system, unlike $CSM\left(\lambda_{ex}^{FC}\right)$ , which will be responsive to variation in the solvent environment. This parameter, therefore, represents entirely novel information over previous models of the REES effect. Specifically, $CSM\left(\lambda_{ex}^{R}\right)$ reports on an extreme of the solvent–fluorophore interaction energy. It can therefore be considered a unique identifying parameter related to both protein structure and physiochemical environment.

The combination of $CSM\left(\lambda_{ex}^{FC}\right)$ and $CSM\left(\lambda_{ex}^{R}\right)$ will therefore be a unique measurement of the accessible equilibrium of protein conformational states and will be specific to a specific protein structure, molecular flexibility, and Trp content and location.

Δ*G* arises from Eq. (4), calculated from the extracted $\lambda_{ex}^{50\%}$ and *m* terms in Eq. (7), where $\lambda_{ex}^{50\%}$ is the $\lambda_{Ex\;}$ at half the maximal CSM and *m* reflects information on the slope of the plot shown in Figure 1C. This gives Δ*G* (J mol^−1^) at a specific wavelength, which has a linear relationship to $\lambda_{Ex\;}$ [Eq. (5)]. For consistency, we report the gradient of the plot of Δ*G* versus $\lambda_{Ex\;}$ , giving Δ*G* expressed in J mol^−1^ nm^−1^, as the value of *m*. Δ*G* reports on the energy gap between adjacent emissive states, for example, in the most extreme case, the gap between the *FC* and *R* states as shown in Figure 1A. As the number of intermediate state increases, reflecting an increased distribution of solvent–fluorophore interaction energies, so the magnitude of Δ*G* will increase, representing a broader distribution of intermediate states.

Inspection of Figure 1A yields two ready predictions for the information content of the parameters in Eq. (7), and we show how these are predicted to affect the resulting experimental data in Figure 1D:

i) A decrease in the gap between $CSM\left(\lambda_{ex}^{FC}\right)$ and $CSM\left(\lambda_{ex}^{R}\right)$ [i.e., an increase in $CSM\left(\lambda_{ex}^{FC}\right)$ ] would reflect a narrower distribution—but unchanged number—of solvent–fluorophore interaction energies. That is, based on Hammond’s postulate (Hammond, 1955), the environments of the *FC* and *R* states become more similar. Experimentally, this would manifest as an increase in the extracted magnitude of $CSM\left(\lambda_{ex}^{FC}\right)$ since $CSM\left(\lambda_{ex}^{R}\right)$ will be a fixed value for a given solvent–fluorophore environment.
ii) In a more rigid molecule, we expect to observe fewer intermediate states. Fewer energetically discrete solvent–fluorophore environments would reflect a larger energy gap between adjacent states (*t*_1_, *t*_2_, etc., Figure 1A). A smaller distribution of solvent–solute interaction energies would manifest as reduced inhomogeneous broadening of the emission spectra (Figure 1C). Experimentally, one then expects a steeper transition between $CSM\left(\lambda_{ex}^{FC}\right)$ and $\;CSM\left(\lambda_{ex}^{R}\right)$ , giving rise to an increased ∆*G*.

Changes in both $CSM\left(\lambda_{ex}^{FC}\right)$ and Δ*G* are possible and indeed likely when studying proteins. As a specific case, for a completely unfolded versus folded protein, we anticipate an increase in $CSM\left(\lambda_{ex}^{FC}\right)$ and an increase in Δ*G*. That is, $CSM\left(\lambda_{ex}^{FC}\right)$ increases due to the increase in solvent exposure of the available Trp residues and Δ*G* increases as the number of intermediate (discrete) solvent–fluorophore interaction energies decreases, tending towards the homogeneous single state where all Trps are completely solvent exposed, i.e., as in (i) where the environments of the *FC* and *R* states become more similar. Clearly “folded” and “unfolded” protein are two extremes of a continuum of states, for example including simple shifts in protein dynamics, molten globule-like states, and partially unfolded states. Figure 1D is not an exhaustive list of anticipated changes but serves to illustrate key examples.

We acknowledge that it is not possible to experimentally reach saturation of the Trp REES effect $\left(CSM\left(\lambda_{ex}^{R}\right)\right)$ using conventional spectrometers owing to the technical limitations of the intensity of UV light (using halogen lamps) and convolution of the emission spectra with the relatively broad-band excitation achieved from monochromation at the large slit widths necessary to increase illumination. In practice, we find that the signal to noise ratio becomes intractable beyond $\lambda_{Ex\;}\approx 310$ nm for the same concentration of protein. We discuss this in more detail below.

### Tryptophan in Solution

**FIGURE 2:**
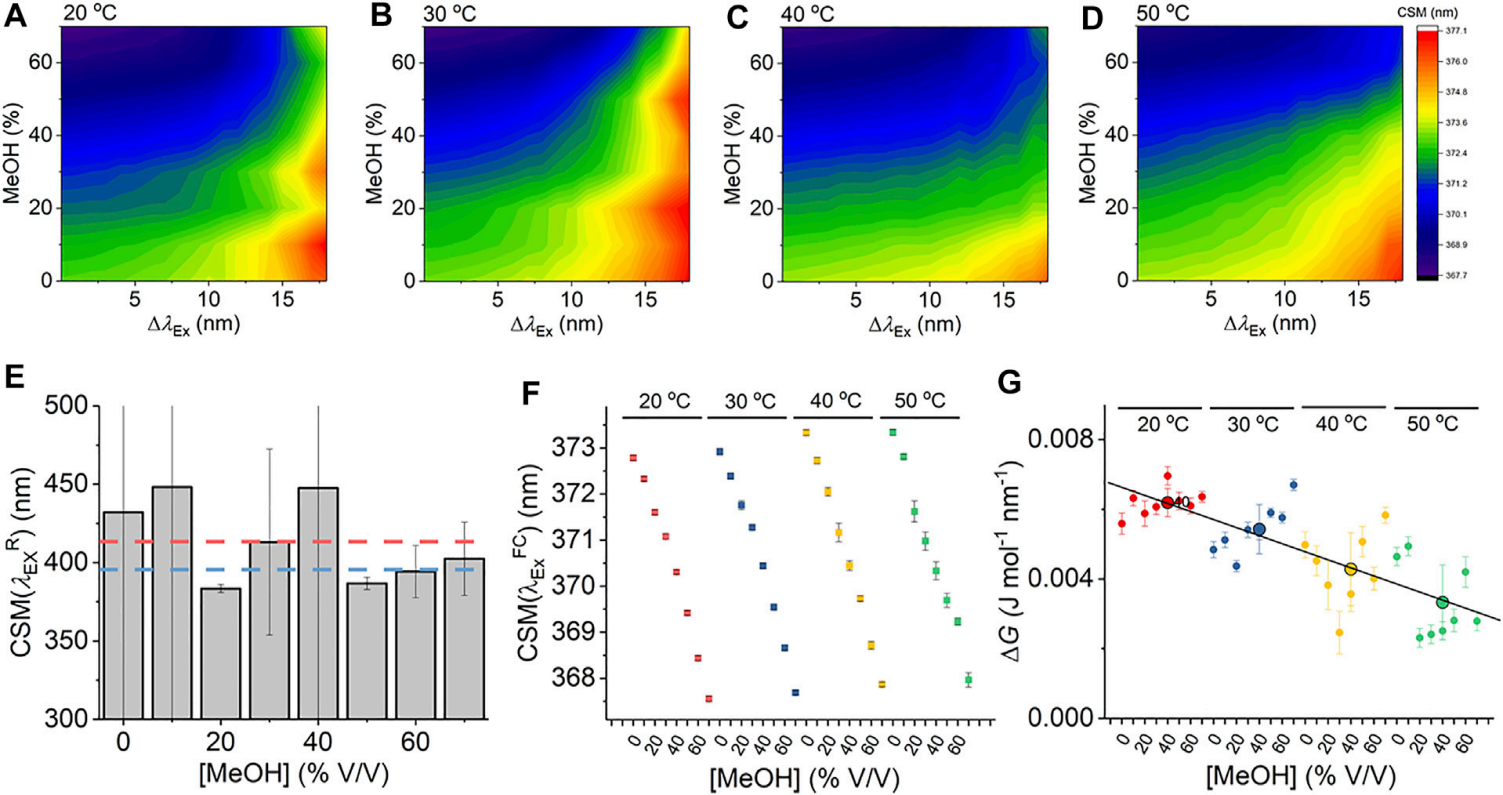
Solvent and temperature studies of the Trp REES effect. **(A–D)** Variation in CSM for L-Trp with varying percentages of MeOH and versus temperature. **(E)** Variation in the $CSM\left(\lambda_{ex}^{R}\right)$ value for each [MeOH] studied, where the fitted $CSM\left(\lambda_{ex}^{R}\right)$ value is a shared parameter for each temperature at a given [MeOH]. The dashed red line shows the average $CSM\left(\lambda_{ex}^{R}\right)$ value, and the blue dashed line shows the $CSM\left(\lambda_{ex}^{R}\right)$ value extracted where all data are fit to Eq. (7) with $CSM\left(\lambda_{ex}^{R}\right)$ as a shared parameter. **(F)** Variation in the $CSM\left(\lambda_{ex}^{FC}\right)$ value for each condition studied. **(G)** Variation in the *ΔG* at fixed *λ*_Ex_ value for each condition studied. Large colored dots represent the average of [MeOH] at each temperature, and the error bars are the standard deviation. Conditions, 1 μM L-Trp, 50 mM Tris–HCl, pH 8.0.

Given that Eq. (7) is a new thermodynamic model for the REES effect, we first explore the sensitivity of the Trp REES effect to variation in the physical properties of the solvent. Solvent studies have been used to probe the sensitivity of the REES effect using viscous matrices such as ethylene glycol and glycerol and temperature variation, by monitoring Trp or indole emission. (Azumi et al., 1976; Demchenko and Ladokhin, 1988). One expects the REES effect to be sensitive to changes in the dielectric constant and viscosity of the solvent and the temperature owing to the effect on the lifetime of solvent relaxation as described above. We are not aware of a method to independently vary dielectric constant, viscosity, and temperature, so we have employed a matrix effect experiment, where we monitor the Trp REES effect as a function of methanol (MeOH) concentration (0%–70% v/v with buffered Tris–HCl, pH 8.0 as in Figure 2) and temperature (20°–50°C). Supplementary Figure S1 shows the variation in viscosity and dielectric constant for the conditions we used. Using this approach, we are able to explore the REES effect, which is quantified using Eq. (6) across a range of conditions. Figures 2A–D show the REES data as a function of the variation in MeOH concentration at each temperature studied. These data are then fit to Eq. (7), and the resulting parameters are shown in Figures 2E–G.

As we describe above, accessing the limiting value of $CSM\left(\lambda_{ex}^{R}\right)$ experimentally is challenging, and thus, the extracted value of $CSM\left(\lambda_{ex}^{R}\right)$ from fits to Eq. (7) will necessarily have a large error, and in some cases, the extracted values are unrealistically large (>1,000 nm). As an alternative, one can share the value of $CSM\left(\lambda_{ex}^{R}\right)$ during fitting, which provides much greater restraint and improved accuracy on the extracted magnitude of $CSM\left(\lambda_{ex}^{R}\right)$ . Fitting with $CSM\left(\lambda_{ex}^{R}\right)$ as a shared parameter for all the data sets gives an average and standard deviation of $CSM\left(\lambda_{ex}^{R}\right)=398\pm 8.0\;\mathrm{nm}$ , respectively (Figure 2E). However, we are aware that this likely masks much of the real variation in the magnitude of $CSM\left(\lambda_{ex}^{R}\right)$ , not least because we expect variation in this parameter with changes in dielectric constant. Alternatively, fitting the data with shared values of $CSM\left(\lambda_{ex}^{R}\right)$ for the same [MeOH] but at varying temperatures (Figure 2E) gives $CSM\left(\lambda_{ex}^{R}\right)=413.5\pm 26.2\;\mathrm{nm}$ . These data suggest a practical range of $CSM\left(\lambda_{ex}^{R}\right)$ (at least across the range of the conditions explored in Figure 2) from ∼387 to ∼440 nm. Supplementary Figure S2 shows modeled data showing the effect of varying $CSM\left(\lambda_{ex}^{R}\right)$ on the extracted magnitude of Δ*G* [there is no effect on $CSM\left(\lambda_{ex}^{FC}\right)$ ]. These data show a ∼10% variance in Δ*G* across the range of $CSM\left(\lambda_{ex}^{R}\right)$ values tested, and so the effect of using a fixed value of $CSM\left(\lambda_{ex}^{R}\right)$ is not large. We note that the range of dielectric constant and viscosity values that this represents is far broader than for a protein in aqueous solvent. Therefore, while not ideal, until it is experimentally possible to extract data at very low excitation energies $\left(>\lambda_{Ex\;}=310\;\mathrm{nm}\right)$ , fixing the magnitude of $CSM\left(\lambda_{ex}^{R}\right)$ is necessary to extract realistic values for Δ*G*, and our data imply that this will not cause a large effect on protein data. We therefore use $CSM\left(\lambda_{ex}^{R}\right)=398.7$ (as above) to extract values of $CSM\left(\lambda_{ex}^{FC}\right)$ and Δ*G* for the data shown in Figures 2F and G.

Figure 2F shows the variation in $CSM\left(\lambda_{ex}^{FC}\right)$ for each [MeOH] at each temperature studied. At all temperatures, the magnitude of $CSM\left(\lambda_{ex}^{FC}\right)$ decreases with increasing [MeOH]. This decrease is expected for a simple solvatochromatic shift and has been observed in numerous cases previously. This expected finding is satisfying because it validates the interpretation of $CSM\left(\lambda_{ex}^{FC}\right)$ value as an excitation wavelength-independent metric of Trp solvation. Supplementary Figure S3 shows the temperature dependence of $CSM\left(\lambda_{ex}^{FC}\right)$ at each [MeOH], extracted from fitting to a simple linear function. Supplementary Figure S3 shows a “V-shaped” temperature dependence with respect to [MeOH], with a minimum at 30% [MeOH], where there is no measurable temperature dependence of $CSM\left(\lambda_{ex}^{FC}\right)$ within error. Therefore, our data suggest that in aqueous solvent, $CSM\left(\lambda_{ex}^{FC}\right)$ appears to have an intrinsic temperature dependence of ∼0.02 nm^−1^ K^−1^ for free Trp in aqueous solution. We consider whether this is borne out in protein samples below. Figure 2G shows the variation in the extracted magnitude of Δ*G* as a function of [MeOH] at each temperature studied. We find a general decrease in the magnitude of Δ*G* with increasing temperature (−0.1 × 10^−3^ J mol^−1^ nm^−1^ K^−1^ across the range studied). Increased temperature will increase *τ*_S_; thus, one anticipates a smaller REES effect and, as described above, a decrease in the magnitude of Δ*G* as we indeed observed. Our data track with a logical and expected physical effect validates the principles used to derive Eq. (7).

From Figure 2G, we do not observe a consistent trend in the magnitude of Δ*G* with respect to [MeOH]. It is not possible to independently vary viscosity, dielectric constant, and temperature, with viscosity having a strong dependence on both temperature and [MeOH]. In contrast to $CSM\left(\lambda_{ex}^{FC}\right)$ , it is evident that Δ*G* is acutely sensitive to such interdependencies. It is therefore not possible to assess simple trends in *ΔG* as a function of [MeOH]. To illustrate this point, we have plotted the magnitude of Δ*G* versus the calculated solvent viscosity and dielectric constant for the combination of [MeOH] and temperature used—Supplementary Figure S1C. From this figure, it is apparent that there is a complex trend governing the magnitude of Δ*G*, resembling an elliptical phase-type relationship. What these data do serve to illustrate is not only the extreme sensitivity of the REES effect to the solvent environment as predicted but also the potential sensitivity of Eq. (7) to track these subtle changes in the distribution of solvent–solute interaction energies. We note that the solvent conditions that we used are not particularly viscous. That we are able to observe a REES effect under these conditions (above) illustrates the sensitivity of the REES effect under less extreme conditions.

**FIGURE 3:**
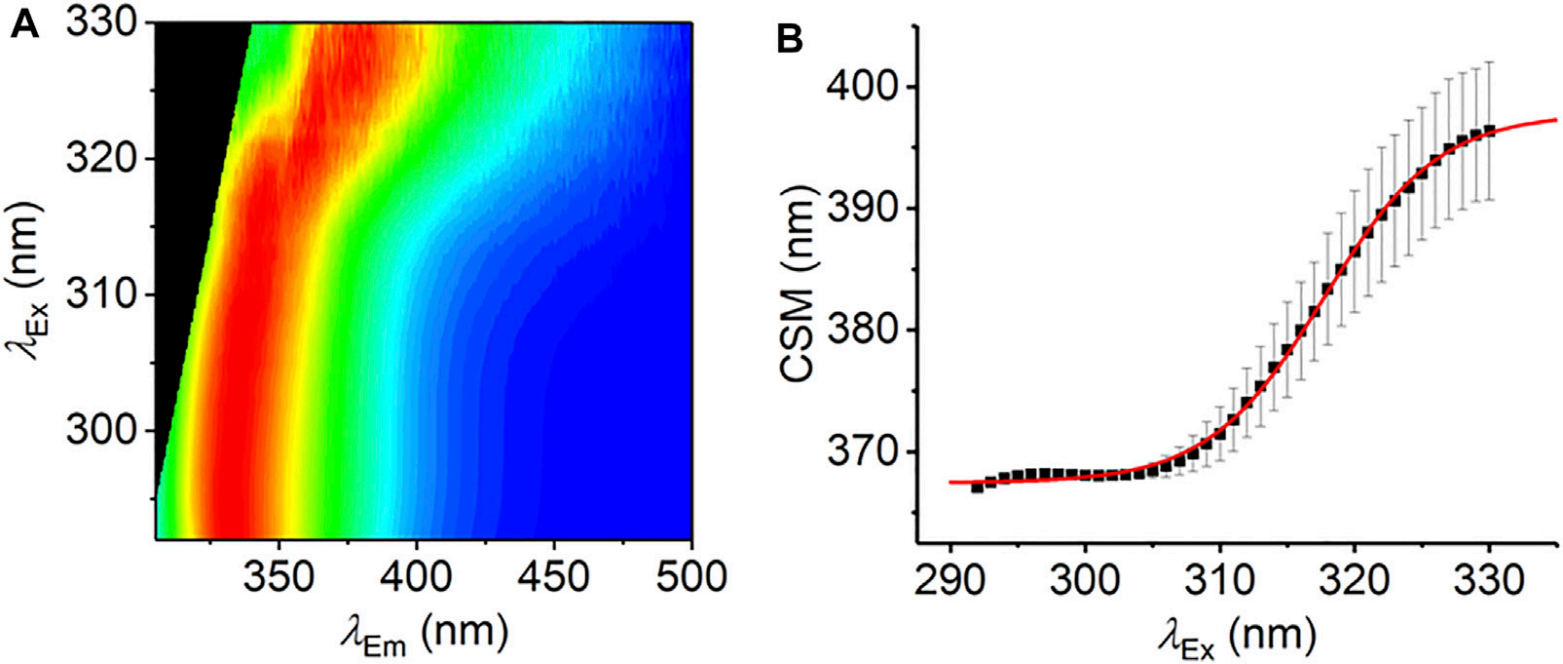
The REES effect monitored over extended *λ*_Ex_ range. **(A)** Averaged, normalized raw emission spectra. **(B)** CSM data extracted from *λ*_Em_ = 340–500 nm. The solid red line is the fit to Eq. (7). Data collected in triplicate; error bars are the standard deviation. Conditions: 1.25 mM L-Trp, MeOH, 25°C.

Our data using free Trp in solution provides a detailed baseline for the sensitivity of Eq. (7) to track the protein Trp REES effect, most notably establishing realistic ranges for the magnitude of $CSM\left(\lambda_{ex}^{R}\right)$ and the temperature dependence of $CSM\left(\lambda_{ex}^{FC}\right)$ and illustrating the extreme sensitivity of the magnitude of Δ*G* to a change in the solvent–fluorophore interaction energies. We wished to directly validate the saturation of CSM $\left(CSM\left(\lambda_{ex}^{R}\right)\right)$ as shown in Figure 1C and to confirm that the extracted value of $CSM\left(\lambda_{ex}^{R}\right)=398.7$ from Figure 2 is an accurate reflection of $CSM\left(\lambda_{ex}^{R}\right)$ for Trp. As we discuss above, there are significant technical challenges in collecting a “complete” REES data set (measuring emission spectra at *λ*_Ex_ > 310 nm). However, using a combination of elevated L-Trp concentration (1.25 mM), a non-aqueous solvent (100% MeOH), and high excitation power (∼100 µW), we have achieved this goal, as shown in Figure 3. Figure 3A shows the averaged raw spectral data. CSM is calculated in the range of 340–500 nm to be consistent across all excitation wavelengths used without being convolved of excitation peaks. From Figure 3B, the resulting CSM data saturate as predicted by Eq. (6) and fitting using Eq. (7) gives $CSM\left(\lambda_{ex}^{R}\right)=397.8\pm 4.0$ . This compares with $CSM\left(\lambda_{ex}^{R}\right)=398.7\pm 8.0\;\mathrm{nm}$ extracted from fitting to the Trp REES data (Figure 2) as described above. That these values are effectively identical is a powerful validation that $CSM\left(\lambda_{ex}^{R}\right)$ extracted from simultaneous fitting of REES data (Figure 2) is accurate and that the high [Trp] used in Figure 3 does not give rise to artifacts, e.g., from homotransfer. To our knowledge, this is the first experimental measurement of a complete REES data set. However, we note that the conditions used (very high concentration and non-aqueous solvent) are not practical for proteins, and we consider alternative routes to achieve this below. That is, the data serve to illustrate that the REES effect saturates as expected and as predicted by our model.

### Single Trp Proteins

**FIGURE 4:**
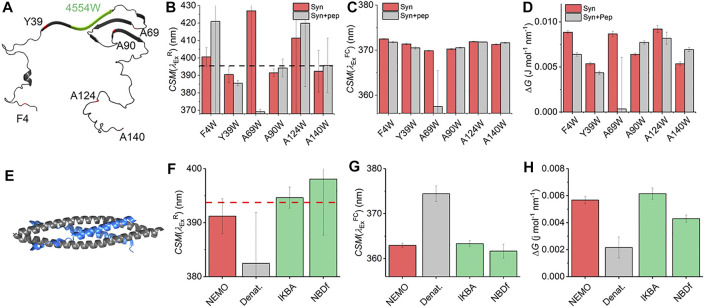
Single Trp protein REES. **(A)** Structural model of α-synuclein (PDB 2n0A24). **(B–D)** Variation in $CSM\left(\lambda_{ex}^{R}\right)$ **(B)**, $CSM\left(\lambda_{ex}^{FC}\right)$ **(C)**, and *ΔG* **(D)** extracted from fits of raw REES data to Eq. (7) for α-synuclein (red bars) and in the presence of the therapeutic peptide (gray bars). The black dashed line in **(B)** shows the $CSM\left(\lambda_{ex}^{R}\right)$ value extracted where all data are fit to Eq. (7) with $CSM\left(\lambda_{ex}^{R}\right)$ as a shared parameter. **(E)** Structural model of the N-terminus of NEMO (PDB 3brv) in complex with a peptide representing IKKβ (blue). Conditions, 5 µM α-synuclein, 50 mM Tris–HCl, pH 8.0. **(F–H)** Variation in $CSM\left(\lambda_{ex}^{R}\right)$ **(B)**, $CSM\left(\lambda_{ex}^{FC}\right)$ **(C)**, and Δ*G* **(D)** extracted from fits of raw REES data to Eq. (7) for NEMO (red bars), under denaturing conditions (gray bars) and in the presence of ligands (green bars). The red dashed line in panel **(F)** shows the $CSM\left(\lambda_{ex}^{R}\right)$ value extracted where all data are fit to Eq. (7) with $CSM\left(\lambda_{ex}^{R}\right)$ as a shared parameter. Raw NEMO REES data as previously reported in Ref. 28.

With the characterization of the REES effect for free Trp in solution in hand, we now turn to single Trp-containing proteins to establish how the REES effect (quantified with Eq. (7)) changes when the Trp is part of a complex polymer (protein). We have selected a large, monomeric (48 kDa; 419 aa) human regulatory protein, which natively has a single Trp [NF-κB essential modulator (NEMO)] (Barczewski et al., 2019), and a natively unstructured protein (α-synuclein, 140 aa) (Meade et al., 2019) that lacks native Trp residues but where we have engineered them into specific sites. These model systems allow us to explore a broad range of conditions and physical environments for single Trp proteins. It also enables us both to explore the sensitivity of Δ*G* and, similar to our Trp in solution studies, define the range of $CSM\left(\lambda_{ex}^{R}\right)$ magnitudes for protein/peptides in an aqueous environment versus the much broader range of physical conditions studied for Trp in MeOH/water mixtures as described above. Figure 4A shows a structural model of α-synuclein, with the location of the selected sites for Trp incorporation. α-Synuclein is thought to be a largely unstructured (lacking secondary structure) monomer, but which organizes into a β-sheet-rich fibrillar-like architecture as a repeating unit with a “Greek Key” motif (Figure 4A) (Meade et al., 2019). The Trp incorporation sites were selected because, in a previous work, they were found not to alter the aggregation propensity of α-synuclein but did show a measurable REES effect (Jain et al., 2013). In addition, we show data for α-synuclein in the presence and absence of a therapeutic peptide (KDGIVNGVKA), designed to prevent aggregation to the toxic species (as we have reported previously) (Jain et al., 2013). This peptide is based on residues 45–54 of the α-synuclein sequence (Figure 4A; green coloration), and therefore, binding will be in that location (Meade et al., 2020), and we do not expect the variants to alter this binding given they are not within this sequence. This peptide has been shown to bind to a partially aggregated form of α-synuclein (Meade et al., 2020). Figure 4B shows the $CSM\left(\lambda_{ex}^{R}\right)$ value extracted from the REES data from independent fits [no shared $CSM\left(\lambda_{ex}^{R}\right)$ value] to each of the α-synuclein variants and in the presence of the therapeutic peptide. The $CSM\left(\lambda_{ex}^{R}\right)$ values vary between ∼385 and ∼425 nm (noting the very large attendant error values in Figure 4B) with an average and standard deviation of $CSM\left(\lambda_{ex}^{R}\right)=400.4\pm 15.4\;\mathrm{nm}$ , respectively. Sharing the value of $CSM\left(\lambda_{ex}^{R}\right)$ during the fitting to Eq. (7) gives $CSM\left(\lambda_{ex}^{R}\right)=395.5\pm 0.1\;\mathrm{nm}$ . It is worth noting these values of $CSM\left(\lambda_{ex}^{R}\right)$ are effectively identical to those extracted for Trp in solution (Figures 2 and 3). For consistency, in our data analysis, we have used $CSM\left(\lambda_{ex}^{R}\right)\;=395.5$ to extract the magnitude of $CSM\left(\lambda_{ex}^{FC}\right)$ and Δ*G*, as discussed above.

Figure 4C shows the extracted $CSM\left(\lambda_{ex}^{FC}\right)$ values for each variant, with and without the therapeutic peptide bound. The magnitude of $CSM\left(\lambda_{ex}^{FC}\right)$ shows variation with Trp position, likely reflecting the combination of the difference in solvent exposure and the immediate electronic environment arising from differences in amino acid composition flanking each Trp. As discussed above, this is effectively a solvatochromatic effect as is typical of Trp emission. However, in the presence of the therapeutic peptide, we find a substantial shift to a lower wavelength for A69W, suggesting a significant decrease in solvent exposure at residue 69 upon peptide binding. Figure 4D shows the resulting Δ*G* values at each site, extracted from fitting the REES data to Eq. (7). We find that the magnitude of Δ*G* varies depending on the specific Trp location in the α-synuclein peptide, which potentially points to some non-globular local structural organization, similar to a molten globule-like protein. Alternatively, the differences might be attributable to the specific amino acid sequence immediately flanking these positions providing a different distribution of solvent–fluorophore interaction energies. Moreover, the addition of the therapeutic peptide decreases the magnitude of Δ*G* most significantly at a single site, residue 69, similar to our findings for $CSM\left(\lambda_{ex}^{FC}\right)$ .

The finding of a decrease in both Δ*G* and $CSM\left(\lambda_{ex}^{FC}\right)$ at AA69 on peptide binding suggests that incubation with the therapeutic peptide decreases solvent exposure and increases flexibility at AA69. From Figure 4A, A69W is the variant that is most structurally localized with the anticipated binding site of the therapeutic peptide (green color in Figure 4A). Therefore, our finding of a decreased solvent exposure and shift in flexibility at AA69 is entirely consistent with the putative binding location and the disruption of the putative Greek key motif. These data are powerful evidence that the REES effect, quantified with Eq. (7), could be used to track ligand binding and specifically protein–protein interactions.

NF-κB essential modulator (NEMO) is a 48 kDa human regulatory protein involved in the mediation of the NF-κB signaling pathway. A range of studies suggest that NEMO is a flexible protein and can undergo ligand-specific conformational change (Catici et al., 2015; Catici et al., 2016). NEMO has a single native Trp residue (W6), which is conveniently located close to the residues that bind to the kinase regulated by NEMO (Figure 4E), IκB kinase-β (IKK-β) (Barczewski et al., 2019). Moreover, there is evidence that the IKK-β substrate, IκBα, is also able to interact with NEMO (Vieille and Zeikus, 2001). We have previously reported the binding of peptide mimics of these proteins to NEMO. We note that the peptides lack Trp residues either natively (IKBα) or by design [NBD-Phe, where the native Trp of the NEMO biding domain (NBD) of IKK-β is replaced by Phe] (Catici et al., 2016). Figures 4F–H show the results of fitting Eq. (7) to NEMO REES data in native and denatured forms and in the presence of these two ligands.

From Figure 4F we find that the extracted magnitude of $CSM\left(\lambda_{ex}^{R}\right)$ is similar for the different conditions that we studied (denatured in 8 M urea and with different ligands bound), although we acknowledge that the attendant error is very large. As with α-synuclein, we fit the combined data to Eq. (7) while sharing the $CSM\left(\lambda_{ex}^{R}\right)$ parameter, which gives $CSM\left(\lambda_{ex}^{R}\right)=394.0\pm 1.3$ . As with α-synuclein, we used this value for $CSM\left(\lambda_{ex}^{R}\right)$ to extract the magnitude of Δ*G* for NEMO.

From Figure 4G, we find that the magnitude of $CSM\left(\lambda_{ex}^{R}\right)$ is similar within error for NEMO with and without ligands bound. However, for the unfolded protein in 8 M urea, we found that $CSM\left(\lambda_{ex}^{R}\right)$ increases from $CSM\left(\lambda_{ex}^{FC}\right)=363\pm 0.5\;\mathrm{to}\;374.4\pm 1.7\;\mathrm{nm}$ . As we discussed above, the magnitude of $CSM\left(\lambda_{ex}^{FC}\right)$ appears to reflect the degree of solvent exposure to the aqueous environment. Therefore, the observation of an increase in $CSM\left(\lambda_{ex}^{FC}\right)$ in the presence of denaturant is consistent with tracking an unfolded form of the protein. Figure 4H shows the magnitude of Δ*G* for denatured NEMO and with ligands bound. These data show a decrease in Δ*G* when NEMO is denatured (ΔΔ*G* = 0.002 ± 0.001 J mol^−1^ nm^−1^), no change outside of error in the presence of IKBα (ΔΔ*G* = 0.006 ± 0.0004 J mol^−1^ nm^−1^), and a slight decrease with NBD-Phe bound (ΔΔ*G* = 0.004 ± 0.0003 J mol^−1^ nm^−1^).

Combined, our data provide a means to interpret the physical meaning of the magnitude of Δ*G*. In the case of the denatured NEMO, the increase in $CSM\left(\lambda_{ex}^{FC}\right)$ reflects the unfolding of NEMO as an increase in aqueous solvent exposure of the single native Trp residue. The observation of a decrease in the magnitude of Δ*G* would seem consistent with a more heterogeneous (less folded) protein. Binding of NBD-Phe similarly decreases the magnitude of Δ*G* but to a much lesser extent than for unfolded NEMO. Moreover, unlike in the case of the unfolded protein, the magnitude of $CSM\left(\lambda_{ex}^{FC}\right)$ is essentially invariant within error. These data would then suggest a structurally similar protein, but with a partially restricted distribution of conformational states, arguably more “folded” than NEMO alone. This inference seems credible since binding of NEMO to IKKβ gives a well-folded α-helical dimer (Figure 4E), despite the binding interface being highly dynamic (Barczewski et al., 2019). Moreover, these findings track with the binding of the therapeutic peptide to α-synuclein, which shows a similar decrease in the magnitude of Δ*G* on ligand binding (discussed above).

NEMO and α-synuclein give similar $CSM\left(\lambda_{ex}^{R}\right)$ values with an average and standard deviation of $CSM\left(\lambda_{ex}^{R}\right)=397\pm 15.2\;\mathrm{nm}$ , respectively (Figures 4B, F), respectively. That is, we find a very similar $CSM\left(\lambda_{ex}^{R}\right)$ from several different single Trp proteins, differing in size, structure, and physical environments (different location in peptide, ligand bound/free). This finding tracks well with our solution Trp studies. We note that the $CSM\left(\lambda_{ex}^{R}\right)$ value is smaller than Trp in solution but not outside of the calculated error. Potentially, the lower $CSM\left(\lambda_{ex}^{R}\right)$ value suggests that Trp in a peptide experiences a restricted range of solvent–solute interaction energies compared to Trp in solution, i.e., Trp in a peptide cannot access emissive states that are as low energy as those in solution. This is a logical conclusion given that Trp in a peptide will necessarily have restricted orientational freedom compared to bulk solvent. However, we stress the large error values on the $CSM\left(\lambda_{ex}^{R}\right)$ values reflecting the anticipated range of potential $CSM\left(\lambda_{ex}^{R}\right)$ values for Trp in peptides.

These data therefore provide a “baseline” range for $CSM\left(\lambda_{ex}^{R}\right)$ , which should reflect a limiting case for the value of $CSM\left(\lambda_{ex}^{R}\right)$ for Trp in a peptide. Fitting all our single protein Trp and solution Trp data to a shared $CSM\left(\lambda_{ex}^{R}\right)$ value gives $CSM\left(\lambda_{ex}^{R}\right)=395.4\pm 0.9\;\mathrm{nm}$ . This value then represents a limiting value for $CSM\left(\lambda_{ex}^{R}\right)$ drawn from a very broad range of solvent–Trp interaction energies; it is effectively an average value. Clearly, using this value of $CSM\left(\lambda_{ex}^{R}\right)$ as a fixed standard for fitting Trp REES data has significant caveats. However, given the challenge of capturing meaningful data at elevated excitation wavelengths and that our modeled data (Supplementary Figure S2) showed that Δ*G* is highly tolerant to variation in $CSM\left(\lambda_{ex}^{R}\right)$ , we have chosen to use this value with the much more complex data sets involving multi-Trp proteins (below). For multi-Trp proteins, the extracted REES effect will be an average across all solvent–Trp environments and so the use of a well-parameterized average value of $CSM\left(\lambda_{ex}^{R}\right)$ is logical. We discuss the potential for experimentally accessing $CSM\left(\lambda_{ex}^{R}\right)$ below.

### Multi-Trp Protein

**FIGURE 5:**
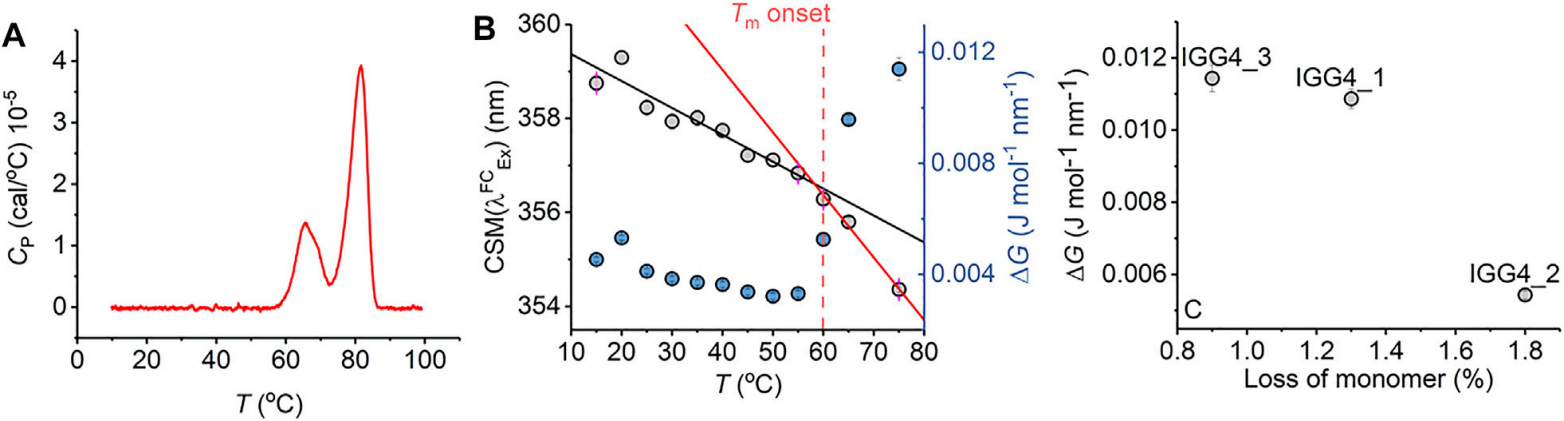
Antibody stability prediction and the effect of temperature. **(A)** Differential scanning calorimetry data for mAb1. **(B)** Temperature dependence of parameters extracted from fitting the IgG1 REES data to Eq. (7). **(C)** Percentage loss of monomer for mAb1-3 after 6 months incubation at room temperature versus Δ*G* extracted from fitting REEs data to Eq. (7) at *t* = 0. Raw REES data from panel **(C)** as reported previously in Ref. 14.

Having established a limiting value of $CSM\left(\lambda_{ex}^{R}\right)$ , we now explore multi-Trp proteins. We have recently demonstrated that the protein REES effect can be used to predict changes in stability of multi-Trp proteins, most notably even for proteins with very large numbers of Trp residues such as monoclonal antibodies (Knight et al., 2020). Example raw spectral data are shown in Supplementary Figure S4. We wish to explore whether Eq. (7) retains this predictive power and to probe its sensitivity. Figure 5 shows the temperature dependence of Δ*G* for a therapeutic mAb (IGg4-based; 150 kDa; 22 Trp residues), which is in commercial development. Figure 5A shows differential scanning calorimetry (DSC) data for the mAb, which shows *T*_*m*_ onset at 60°C, followed by two separate unfolding transitions at 67.2°C and 82.9°C. The data shown in Figure 5B are the result of fitting the REES data to Eq. (7) using $CSM\left(\lambda_{ex}^{R}\right)=395.4$ as discussed above. From this figure, we find that as the temperature increases, Δ*G* decreases approximately linearly to ∼60°C (red dashed line) and with an approximately invariant $CSM\left(\lambda_{ex}^{FC}\right)$ within the error of the measurement. This temperature tracks with the identified *T*_m_ onset from the DSC data (Figure 5A). At >60°C, we find that $CSM\left(\lambda_{ex}^{FC}\right)$ increases from 354.3 ± 0.1 at 55°C to 359.1 ± 0.2 at 75°C. This increase in $CSM\left(\lambda_{ex}^{FC}\right)$ is accompanied by a larger decrease in Δ*G*, with ΔΔ*G* = 0.0042 J mol^−1^ nm^−1^ between 55°C and 75°C, compared to ΔΔ*G* = 0.0032 J mol^−1^ nm^−1^ between 15°C and 55°C. That is, we observed a breakpoint in the temperature dependence of Δ*G* (shown as the solid fitted lines). For the 15–55°C range, we found that the temperature dependence of Δ*G* is −0.1 × 10^−3^ J mol^−1^ nm^−1^ K^−1^, precisely as we found for the Trp in solution (Figure 2G). For the 55°C –75°C range, this value becomes larger, –0.25 × 10^−3^ J mol^−1^ nm^−1^ K^−1^. Thus, as the protein unfolds, we find an increase in $CSM\left(\lambda_{ex}^{FC}\right)$ and a decrease in Δ*G*, exactly as with the chemically denatured NEMO (above). These data therefore demonstrate the sensitivity of the protein REES effect, fitted using Eq. (7), to altered conformational states.

Notionally, changes in the equilibrium of conformational states should track with protein stability. That is, as the free energy landscape flattens, more discrete conformational states become accessible (i.e., a broader equilibrium of conformational states), including those corresponding to non-native conformations. For highly structurally similar proteins, we therefore anticipate that a decrease in the magnitude of Δ*G* will correlate with a less thermodynamically stable protein. Figure 5C shows the magnitude of Δ*G* for three monoclonal antibodies, in active development and all based on a common scaffold (IgG4), in relation with the fractional loss of monomer over 6 months at room temperature (reported recently; Ref. 16). From Figure 5C, we find that a decrease in the magnitude of Δ*G* correlates with a decrease in protein stability (as predicted). These data, therefore, suggest that the magnitude of Δ*G* is sensitive not only to the very earliest stages of protein unfolding but also to differences in thermodynamic stability.

**FIGURE 6:**
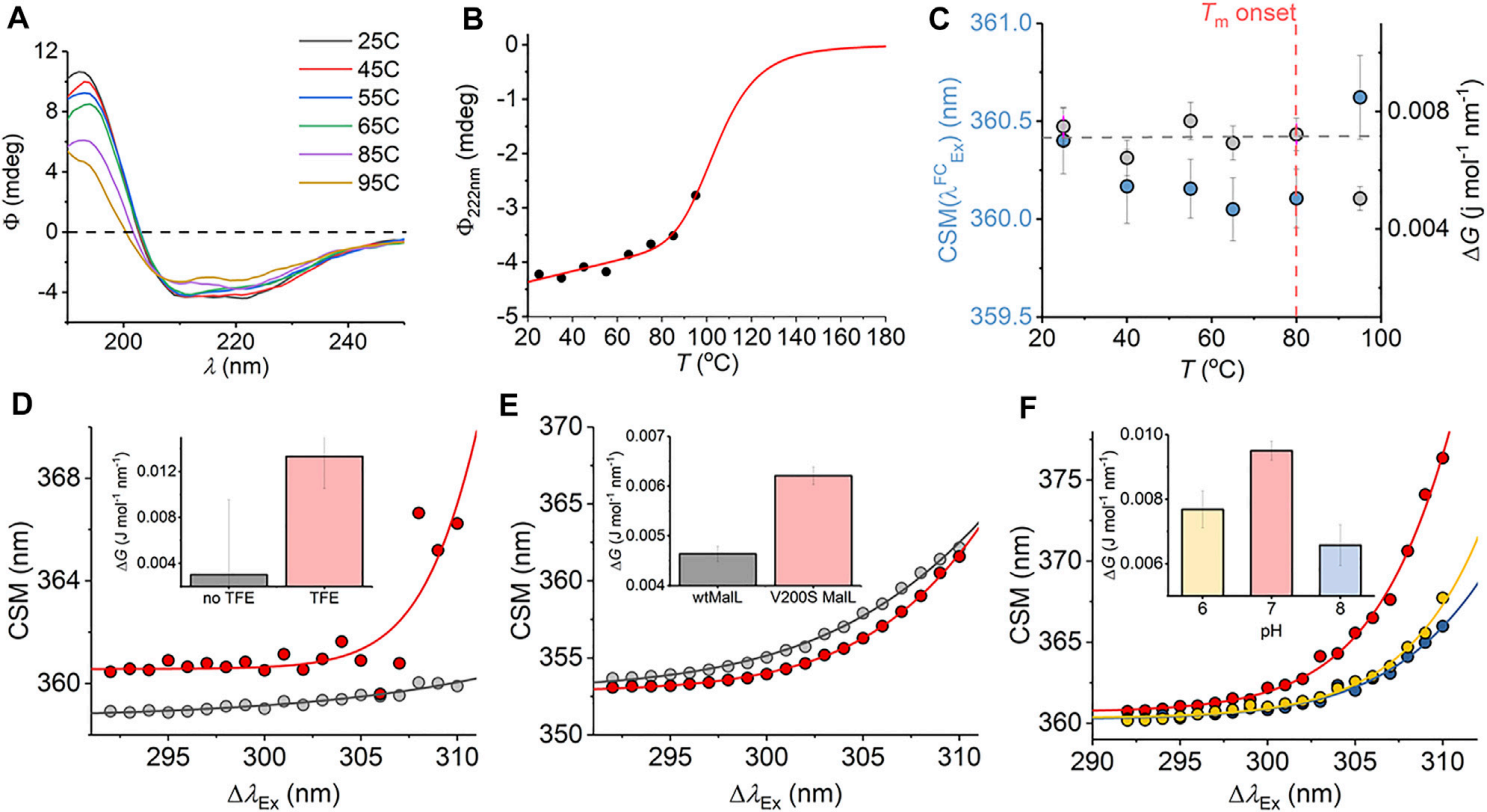
**(A–C)** Temperature dependence of the *ss*GDH REES effect and correlation with unfolding. **(A)** Temperature dependence of far-UV CD spectra. **(B)** Temperature dependence of Φ_222nm_. Solid line is the fit to Eq. (8) as described in the main text. **(C)** Temperature dependence of parameters extracted from fitting the *ss*GDH REES data to Eq. (7) **(D–F)** REES effect of C45 in the presence and absence of TFE, raw data as Ref. 17 **(D)**; *wt*MalL and V200S MalL, raw data as Ref. 16 **(E)**; and *ss*GDH at different pH values **(F)**. The inset bar charts **(D–F)** show the magnitude of Δ*G* extract from fitting to Eq. (7).

We have explored a similar temperature relationship with the hyperthermophilic, tetrameric, glucose dehydrogenase from *Sulfolobus solfataricus* (*ss*GDH). The natural operating temperature of the *S*. *solfataricus* is ∼77°C; *ss*GDH is extremely thermally stable even at elevated temperatures and shows very high rigidity relative to a comparable mesophilic protein. (Vieille and Zeikus, 2001). Figure 6A shows the far-UV circular dichroism data for *ss*GDH at a range of different temperatures. From this figure, there is some change in helical content with respect to temperature, most noticeable from the spectra at >85°C. Figure 6B shows the change in ellipticity at 222 nm (*Φ*_222nm_) with respect to temperature. The solid red line in Figure 6B shows the fit to

$$\theta_{222nm}=\frac{b_{f}+a_{f}T+\left(b_{u}+a_{u}T\right)K_{u}}{1+K_{u}}$$ (8)

where

$$K_{u}=exp\left(\Delta H\left(1-T/T_{m}\right)/RT\right)$$ (9)

where *a* and *b* are the slope and intercept of the folded (*f*) and unfolded (*u*) baseline, respectively. *T*_m_ is the melting temperature, and Δ*H* is the van’t Hoff enthalpy of unfolding at *T*_m_. From Figure 6B, there is no evident complete unfolding transition, and so we have restrained the parameters in Eq. (8) to give a sense of where the unfolding transition would occur and an indicative *T*_m_. That is, we fixed the ellipticity and gradient of the “unfolded” limb of the slope to zero, which is a reasonable approximation. Fitting the data using Eq. (8) gave *T*_m_ = 105.5 ± 5.5°C. That is, the data fits to an unfolding transition that is at an experimentally inaccessible temperature. We note the significant linear slope of the “folded” limb of Figure 6B. This linear phase of the thermal melt does not reflect unfolding, and there is no clear consistent interpretation of the magnitude of *a*_*f*_; it is essentially always removed from analysis (Fenner et al., 2010) Potentially, it reflects changes in solvent dynamics with respect to temperature or more trivial effects. The transition from this linear phase to the apparent unfolding transition is at ∼ 80°C.

From Figure 6C, we find that the magnitude of Δ*G* is essentially invariant with respect to temperature (within the error of the extracted value) up to 80°C. As with mAb1, $CSM\left(\lambda_{ex}^{FC}\right)$ shows a small decrease with respect to temperature to 80°C (<0.5 nm). As the notional unfolding transition occurs (95°C), Δ*G* decreases and $CSM\left(\lambda_{ex}^{FC}\right)$ decreases. These trends are consistent with our observations with mAb1 above. However, *ss*GDH does not show the same decrease in Δ*G* with respect to temperature below the start of the unfolding transition as was evident with mAb1 and also from the anticipated temperature dependence of Δ*G* from our solution Trp studies (Figure 2G). This finding implies that while we anticipate that the Trp REES effect will be temperature dependent, it will be protein specific. Our data do not suggest an immediate physical model for the temperature dependence of the REES effect in different proteins. However, our data potentially point to a more rigid protein (*ss*GDH vs mAb1) having a less temperature-dependent Δ*G* at temperatures below any unfolding transition. The hypothesis that more rigid protein will have a less temperature-dependent REES effect seems logical given our findings of the sensitivity of the protein REES effect to even subtle changes in the equilibrium of protein conformational states.

We were able to more directly explore the trend in Δ*G* on changes in molecular flexibility by correlating with evidence from NMR, simulation, and pH variation. We have recently demonstrated that a *de novo* heme peroxidase (C45; four α-helix bundle; 3 Trp residues) can be rigidified (and stabilized) in the presence of 2,2,2-trifluoroethanol (TFE) (Hindson et al., 2021). The NMR spectra (^1^H-^15^N TROSY-HSQC) show an increase in the number and sharpness of peaks in the presence of TFE, which is indicative of a more rigid protein (Hindson et al., 2021). This rigidification also tracks with an increase in thermal stability (Hindson et al., 2021). Fitting the REES data to Eq. (7) (shown in Figure 6D) gives a Δ*G* value that is measurably larger outside of error in the presence of TFE, Δ*G* = 0.003 ± 0.001 and 0.013 ± 0.004 J mol^−1^ nm^−1^ in the absence and presence of TFE, respectively.

For our multi-Trp examples above, we were not able to rule out conformational change convolved with changes in rigidity/flexibility. Maltose-inducible α-glucosidase (MalL) has become a paradigmatic enzyme for studying the temperature dependence of enzyme activity. (Hobbs et al., 2013). A single amino acid variant (V200S) is sufficient to increase the optimum temperature of reaction (*T*_opt_) from 58°C to 75°C, having an unfolding transition at a higher temperature (Hobbs et al., 2013). Molecular dynamics simulation show that V200S is globally more rigid than the wild-type (*wt*) enzyme, despite the X-ray crystal structures being essentially identical (Hobbs et al., 2013). Therefore, by using MalL we are able to explore the effect of changes in protein rigidity alone on the REES effect. Fitting the extracted REES data to Eq. (7) (shown in Figure 6E) gives a Δ*G* value that is measurably larger outside of error for V200S MalL, Δ*G* = 0.006 ± 0.0002, than for *wt*MalL, 0.004 ± 0.0002 J mol^−1^ nm^−1^.

Finally, we have explored pH variation with *ss*GDH. From our temperature studies (Figures 6A–C), we find that *ss*GDH is extremely structurally stable. In an effort to perturb the stability of *ss*GDH we have explored pH variation. Figure 6F shows the resulting REES data fit to Eq. (7) for *ss*GDH incubated at pH 6, 7, and 8. From Figure 6F inset, we find that the magnitude of Δ*G* is largest at pH 7, with a rather lower values at pH 6 and lowest at pH 8. From our data with the mAb1, C45, and MalL, we find that a larger magnitude of Δ*G* suggests a less flexible protein. Supplementary Figure S5 shows the pH dependence of the dynamic light scattering (DLS) profile. From these data, we cannot identify any oligomeric change associated with pH variation. However, the DLS profiles show some variation in width, which might suggest a shift in the distribution of conformational states. These data do not obviously correlate with our REES data (Figure 6F), but potentially highlight the sensitivity of the REES data to capture changes in the equilibrium of conformational states, which would not otherwise be obvious.

In summary, our combined data with multi-Trp proteins (mAb1, *ss*GDH, C45, and MalL) are consistent with the finding that a decrease in the magnitude of Δ*G* is associated with an increase in flexibility. Moreover, and as expected, reductions in molecular flexibility are correlated with increased stability. Finally, *via* the change in the $CSM\left(\lambda_{ex}^{FC}\right)$ term, we are able to use the fitting to Eq. (7) to separately differentiate changes in molecular flexibility with unfolding. Our data therefore suggest that the REES effect is potentially highly sensitive to changes in molecular flexibility outside of conformational change, as with our findings from MalL. These data therefore point to the sensitivity of monitoring the protein REES effect in multi-Trp proteins, quantified using Eq. (7).

## Conclusion

The REES effect is a drastically underutilized analytical tool, given its potential to sensitively track changes in protein microstates. Developing the theoretical models used to understand the effect has high potential to enable the REES effect to be used for unique applications in protein and biomolecular analysis. For example, Kabir *et al.* have recently posited a model for tracking the REES effect of a fluorescent ligand, potentially enabling the dissection of “hidden” ligand bound states of proteins (Kabir et al., 2021). Furthermore, we have demonstrated that quantifying the REES effect with Eq. (7) potentially allows for prediction of mAb stability, and this has potential for increasing the speed of drug development (Knight et al., 2020).

Our data suggests that the model presented here (Eq. (7)) represents a practically applicable, sensitive framework for quantifying the protein REES effect, based on fundamental thermodynamic theory. Specifically, we find that the magnitude of Δ*G* is sensitive to changes in molecular dynamics without structural change of the protein and specifically appears to be sensitive to changes in protein conformational sampling. Moreover, *via* the additional information provided by the $CSM\left(\lambda_{ex}^{FC}\right)$ term, the model appears sensitive to early stage unfolding events and shows predictive power in assessing protein stability. We anticipate Eq. (7) could be modified to account for known numbers and locations of Trp residues (such as solvent accessible surface area and local protein molecular dynamics). Such data could be incorporated in Eq. (7), e.g., as a weighting criterion to enable Δ*G* to be used as an independent metric of stability. Furthermore, with the advent of a large number of high-resolution protein structures, there is very high scope for the use of homology models to fulfill this purpose where specific structures are not available. We also see scope for applying this model to extrinsic fluorophore probes, and we suggest that solvent studies similar to those we report in Figure 1 will be a valuable starting point to establish $CSM\left(\lambda_{ex}^{R}\right)$ .

**FIGURE 7:**
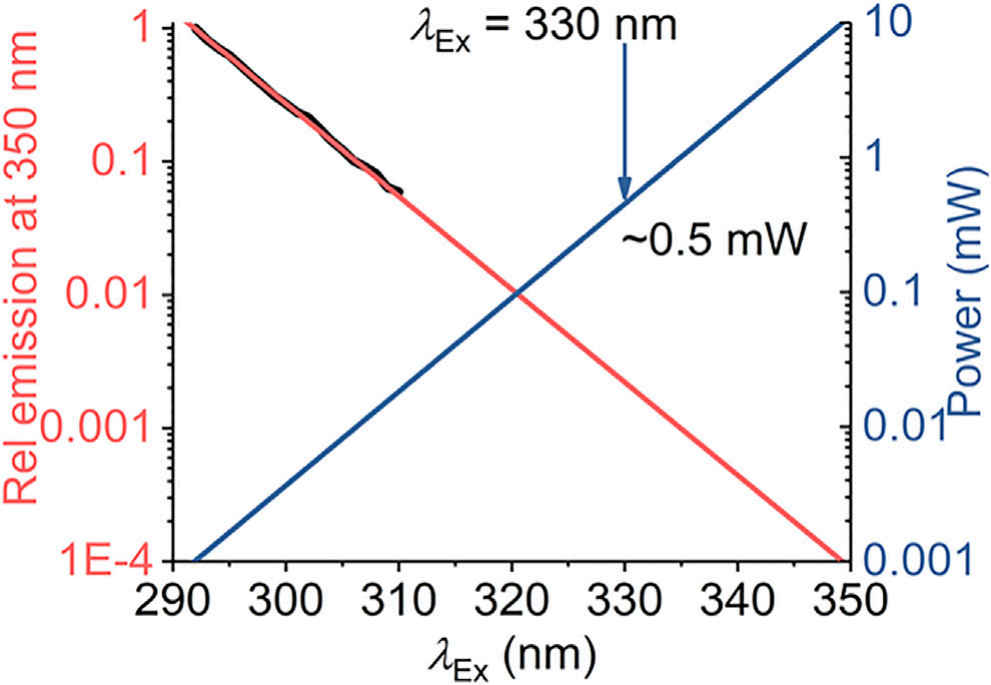
Calculated excitation power requirements to extend protein REES measurements to *λ*_Ex_ > 310 nm. The black line is the experimentally extracted (natural logarithm) excitation spectrum of protein Trp (single Trp of NEMO as Ref. 15). The red line is the fit to a linear function. The blue line is the calculated power required to achieve an equivalent emission intensity at increasing values of *λ*_Ex_.

Our model defines a maximum red shift for a given system, $CSM\left(\lambda_{ex}^{R}\right)$ , which is determined by the fluorophore and its environment. Practically, there is challenge in monitoring a low signal to noise emission spectrum at the elevated excitation wavelengths required to approach $CSM\left(\lambda_{ex}^{R}\right)$ (>∼310 nm), based on the range identified from our experiments. Figure 7 shows modeled power requirements to achieve an equivalent intensity emission signal. From Figure 7, the power requirement is effectively an exponential increase. That is, to accurately characterize $CSM\left(\lambda_{ex}^{R}\right)$ would require ∼0.5 mW at λ_Ex_ = 330 nm. We note that the typical output of commonly used monochromated flash lamps is ∼µW. However, with the rapid development and commercial availability of high-power, stable UV LEDs, high-intensity two/three-photon laser excitation, and laser-driven UV light sources, we anticipate that this should be practically possible.

## Methods

### Red Edge Excitation Shift Data Collection

All fluorescence measurements were performed using a Perkin Elmer LS50B Luminescence Spectrometer (Perkin Elmer, Waltham, MA, United States), an Agilent Cary Eclipse fluorescence spectrometer (Agilent, Santa Clara, CA, United States), or an Edinburgh Instruments FS5 fluorescence spectrometer (data in Figure 3; Edinburgh Instrument, Livingstone, United Kingdom) connected to a circulating water bath for temperature regulation (1°C). Samples were thermally equilibrated by incubation for 5 min at the given conditions prior to recording measurements. Emission spectra were collected for increasing increments of excitation wavelength from 292 nm upwards with increments of 1 nm. The emission spectra were typically collected and analyzed across the range of 325–500 nm to prevent first- and second-order artifacts. Typical slit widths were 5 nm in each case (1.5 nm in the case of the data in Figure 3). For all samples, the corresponding buffer control was subtracted from the spectra for each experimental condition. REES data were collected as described previously (Knight et al., 2020). Data were processed as described in the text by first extracting the CSM values (Eq. (2)) and then fitting with Eq. (6). Data were composed of three to five replicates.

### CD and Dynamic Light Scattering Data Collection

CD data were collected on an Applied Photophysics circular dichroism spectrometer. Corresponding buffer baselines were subtracted for each measurement. DLS data were collected on a Malvern Panalytical Zetasizer using a 50 μl quartz cuvette, thermostated to 25°C.

### Protein Preparation

α-Synuclein, *ss*GDH, and mAb1 were expressed and purified as described previously in Refs. 28, 18, and 16, respectively.

## Data Availability

The original contributions presented in the study are included in the article/Supplementary Material. Further inquiries can be directed to the corresponding authors.
