# Supplementary material for: A Thermodynamic Model for Interpreting Tryptophan Excitation-Energy-Dependent Fluorescence Spectra Provides Insight Into Protein Conformational Sampling and Stability
Source: Front Mol Biosci. 2021 Dec 3;8:778244. doi: 10.3389/fmolb.2021.778244 (PMC8681860; doi:10.3389/fmolb.2021.778244)
Supplement: Supplementary file 1 [file DataSheet1.docx]

Supporting Information. A thermodynamic model for interpreting tryptophan excitation-energy-dependent fluorescence spectra provides insight into protein conformational sampling and stability

Kwok A,^1‡^ Camacho IS,^2‡^ Winter S,^1^ Knight M,^3^ Meade RM,^1^ Van der Kamp MW,^4^ Turner A,^3^ O’Hara J,^3^ Mason JM,^1^ Jones AR,^2^* Arcus, VL,^5^* Pudney CR^1,6^*

1Department of Biology and Biochemistry, University of Bath, BA2 7AY, UK. 2Biometrology, Chemical and Biological Sciences Department, National Physical Laboratory, Teddington, TW11 0LW, UK. 3UCB, Slough, SL1 3WE UK. 4School of Biochemistry, University of Bristol, BS8, 1TD, UK. 5School of Science, Faculty of Science and Engineering, University of Waikato, Hamilton 3240, New Zealand. 6BLOC Laboratories Limited, Bath, BA2 7AY, UK.

**Figures**


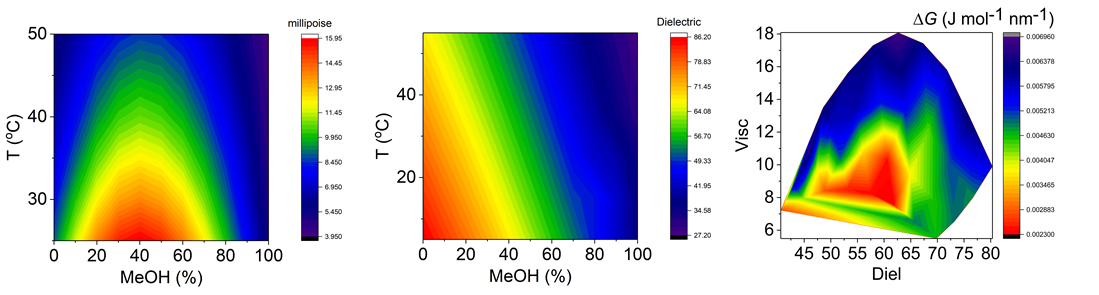


A

B

C

**Figure S1**. **A** and **B**, Variation in viscosity and dielectric on varying MeOH and temperature. **C**, combined viscosity and dielectric dependence of Δ*G*.


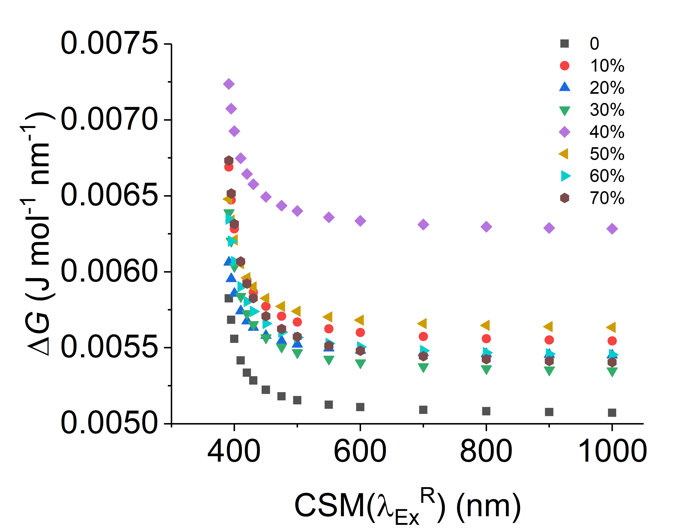


**Figure S2**. Dependence of Trp Δ*G* on $CSM\left( \lambda_{ex}^{R} \right)$ at different MeOH concentrations (20 °C).


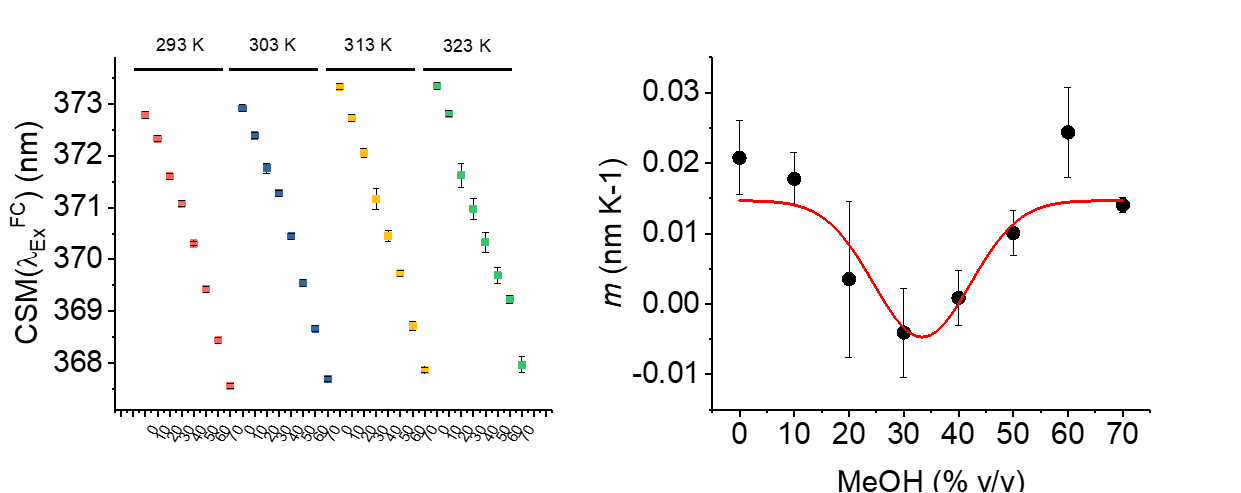


**Figure S3**. Temperature dependence of $CSM(\lambda_{ex}^{FC})$ at different MeOH concentrations from Figure 2F. Where, *m* is the gradient of the fit of $CSM(\lambda_{ex}^{FC})$ *versus*  *T* to a simple linear function.


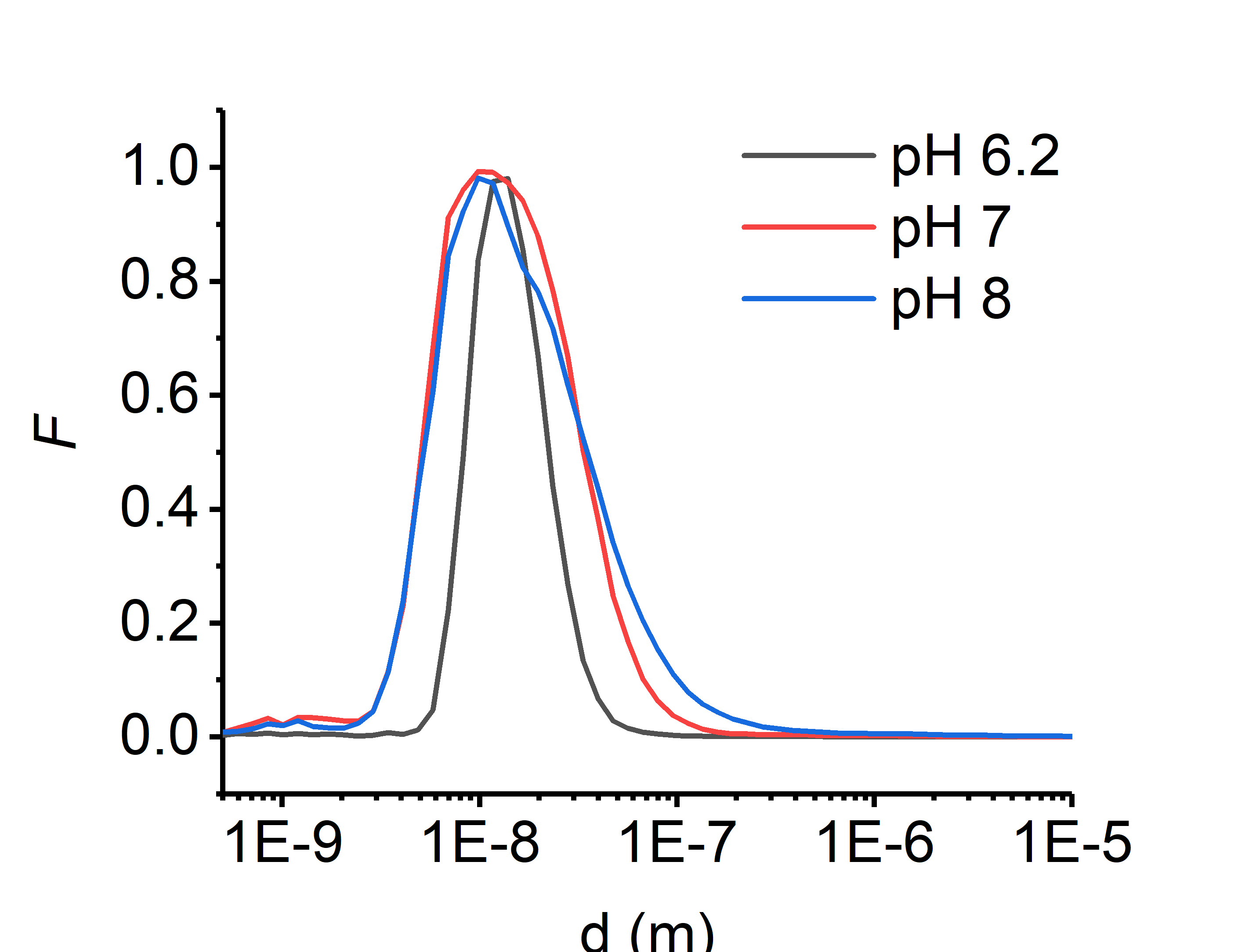


**Figure S4**. Dynamic light scattering profiles for *ss*GDH incubated at different pH values.
